# Spatial Distribution of Senescent Cells and Their Proximity to Immune Subsets in the Human Endometrium During the Implantation Window

**DOI:** 10.3390/diagnostics15212679

**Published:** 2025-10-23

**Authors:** Dimitar Parvanov, Rumiana Ganeva, Margarita Ruseva, Maria Handzhiyska, Jinahn Safir, Lachezar Jelezarsky, Nina Vidolova, Dimitar Metodiev, Georgi Stamenov, Savina Hadjidekova

**Affiliations:** 1 Department of Research, Nadezhda Women’s Health Hospital, 1373 Sofia, Bulgaria; rum.ganeva@gmail.com (R.G.); jelezarsky@gmail.com (L.J.);; 2 Department of Clinical Pathology, Nadezhda Women’s Health Hospital, 1373 Sofia, Bulgaria; 3 Department of Obstetrics and Gynecology, Nadezhda Women’s Health Hospital, 1373 Sofia, Bulgaria; 4 Department of Medical Genetics, Medical University of Sofia, 1431 Sofia, Bulgaria; svhadjidekova@medfac.mu-sofia.bg

**Keywords:** endometrium, senescence, p16, spatial distribution, immune cells, implantation window

## Abstract

**Background/Objectives**: Senescent cells contribute to endometrial remodeling during the implantation window, but their spatial organization within the stroma remains poorly understood. This study aimed to characterize the distribution of senescent (p16-positive) cells in the functional layer of the endometrium and to evaluate their spatial relationships with immune cell subsets. **Methods**: Endometrial biopsies from 68 women undergoing IVF were collected during the mid-luteal phase (LH+7, corresponding to the implantation window). Samples were analyzed by immunohistochemistry for p16 and immune markers (CD3, CD4, CD8, CD14, CD68, CD56, CD79α). Images from adjacent serial sections were digitally aligned, and senescent cell density, clustering, and nearest-neighbor distances to immune cells were quantified using HALO Image Analysis software (v3.4). Ratios of senescent-to-immune cell abundance were also calculated to account for stromal variability. **Results**: Senescent cells were heterogeneously dispersed within the stroma, with occasional high-density clusters. Quantitative analysis revealed that their abundance was lower than that of monocytes, macrophages, and total T cells, but higher than that of T-helper and B cells. Across patients, median senescent-to-immune cell ratios were approximately 1, indicating comparable abundances, except for CD4^+^ and CD79α^+^ subsets, where ratios were significantly elevated. Nearest-neighbor analysis showed that macrophages and monocytes localized in closest proximity to senescent cells (45 ± 20 μm and 45 ± 25 μm), while T-helper and NK cells were positioned at greater distances from senescent cells (102 ± 42 μm and 53 ± 23 μm, respectively). B cells showed the greatest separation (211 ± 66 μm). Correlation analysis confirmed density-driven proximity for most immune subsets, with CD4^+^ and CD56^+^ cells as exceptions, displaying limited spatial association with senescent cells. **Conclusions**: Senescent cells in the endometrium during the implantation window display heterogeneous distribution and selective spatial associations with immune subsets. Their preferential distancing from T-helper and NK cells suggests impaired local immune–senescence crosstalk, highlighting spatial profiling of senescent cells as a potential diagnostic marker of endometrial receptivity.

## 1. Introduction

Embryo implantation requires a receptive endometrium and finely tuned communication between maternal tissues and the embryo. The endometrium is a dynamic and heterogeneous tissue composed of stromal and epithelial cells, vasculature, immune populations, and senescent cells, all of which contribute to the local microenvironment [1,2]. Among these, senescent cells have gained increasing attention as regulators of tissue remodeling and immune modulation. Cellular senescence is characterized by irreversible growth arrest and secretion of bioactive molecules through the senescence-associated secretory phenotype (SASP), which can influence local inflammation, immunity, and extracellular matrix organization [3]. Physiological induction of senescence during decidualization is considered part of normal endometrial renewal, but excessive or spatially dysregulated senescence has been linked to impaired receptivity and infertility [4,5]. Notably, these processes occur within the implantation window, a critical period during which the endometrium acquires receptivity, making the spatial balance between senescent and immune cells particularly relevant for embryo implantation.

Recent studies highlight the active role of immune cells in controlling endometrial senescence. Uterine natural killer (uNK) cells have been shown to eliminate senescent decidual cells, thereby maintaining homeostasis and sustaining the implantation window [4,6]. This immune–senescence crosstalk appears to be tightly regulated by transcription factors such as FOXO1 and has been implicated in recurrent implantation failure and pregnancy loss when dysregulated [6,7]. While these observations point to the biological relevance of senescent–immune interactions, most prior studies have focused on cell abundance rather than their spatial organization. However, the precise spatial dynamics between senescent and immune cells may represent a critical element of endometrial regulation. Aberrant localization or inefficient clearance of senescent cells could disrupt immune surveillance, prolong local inflammation, and compromise the establishment of a receptive microenvironment required for embryo implantation [4,6]. Such mechanisms may underlie the impaired decidual remodeling and implantation failure described in women with dysregulated senescence during the implantation window [7].

Emerging evidence from spatial biology demonstrates that tissue function depends not only on the quantity of cell populations but also on their relative positioning. Multiplexed imaging and computational analysis have shown that proximity and distribution patterns can shape immune function and disease progression [8,9]. In the endometrium, spatial approaches are beginning to be applied to study stromal–immune interactions and may uncover diagnostic biomarkers of receptivity [10,11]. Previous studies, including our own, have suggested that the abundance and ratios of specific immune subsets in the endometrium are associated with implantation success [12,13]. However, the spatial distribution of senescent cells within the endometrial stroma, and their relationships with immune subsets, remains poorly understood. We hypothesized that senescent p16-positive cells display heterogeneous distribution patterns and selective spatial associations with immune populations. Therefore, the aim of this study was to characterize the distribution of senescent cells in the functional layer of the human endometrium and to quantify their proximity to major immune subsets using high-resolution digital image analysis.

## 2. Materials and Methods

### 2.1. Study Design and Participants

This retrospective study was conducted at a private fertility center. Endometrial biopsies were obtained between February 2021 and November 2024 from 68 women undergoing IVF treatment. All procedures were performed in accordance with the Declaration of Helsinki and were approved by the hospital’s Ethics Committee (project code 45/7 December 2020). Written informed consent was obtained from all participants.

Inclusion criteria were as follows: age 32–46 years, regular ovulatory cycles, and no hormonal therapy within the preceding three months. Exclusion criteria were as follows: postmenopausal status, acute or chronic inflammatory disease, antibiotic treatment within the previous three months, endocrinological or genetic disorders, autoimmune disease, oncological disease (including endometrial carcinoma), moderate or severe endometriosis, adenomyosis, uterine hyperplasia, endometrial polyps, or the presence of fibroids. Biopsies were uniformly collected during the mid-luteal phase (LH+7), corresponding to the predicted implantation window.

**Figure 1:**
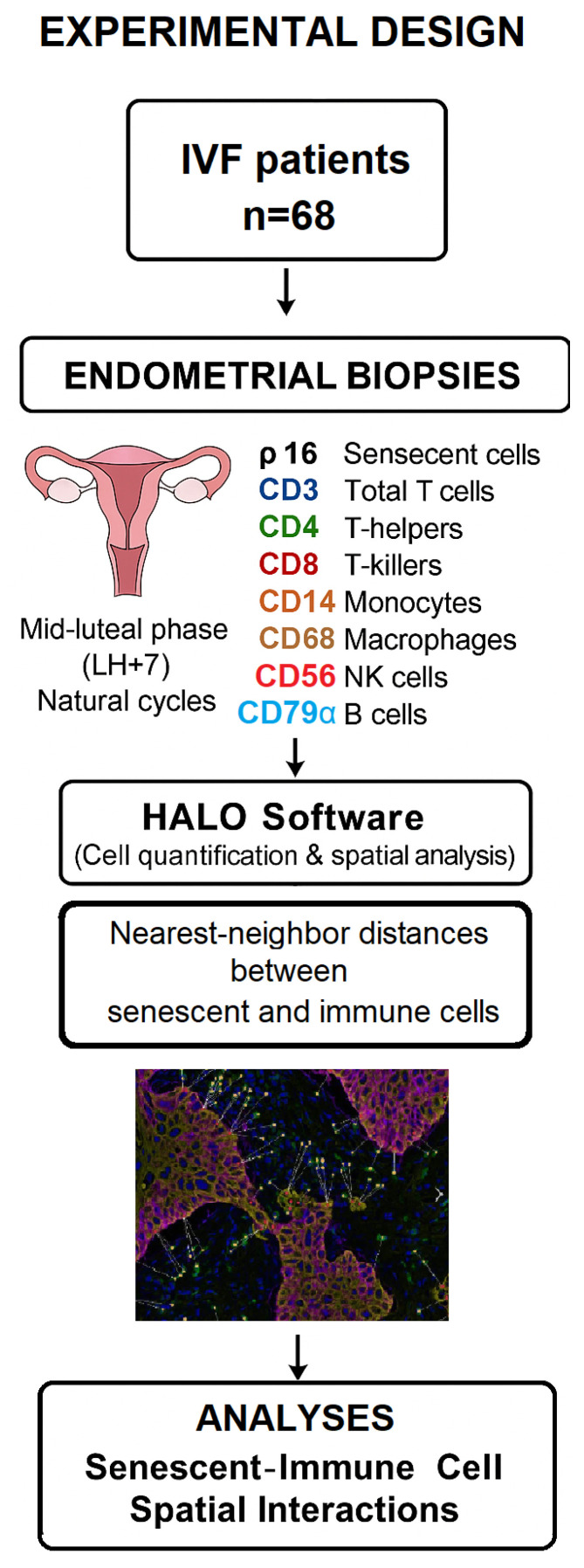
Study experimental design.

The overall experimental workflow, including patient recruitment, endometrial biopsy collection, immunohistochemical staining, and spatial analysis, is summarized in Figure 1.

### 2.2. Tissue Collection and Processing

Endometrial biopsies were collected during the mid-luteal phase (LH+7), corresponding to the predicted implantation window. Tissue samples were fixed overnight in 10% neutral buffered formalin at 4 °C, dehydrated, and paraffin embedded. Serial sections of 4 μm thickness were cut for immunohistochemical staining.

### 2.3. Immunohistochemistry

Immunohistochemistry was performed using the Novolink Polymer Detection System (Leica Biosystems, Newcastle, UK) as previously described [14]. Sections were incubated with a mouse monoclonal antibody against human p16^INK4a^ (MAD-000690QD-7, Master Diagnostica, Madrid, Spain) to identify senescent cells. For spatial proximity analysis, additional sections were stained with rabbit monoclonal or polyclonal antibodies against the following immune markers: CD3 (IS50330-2, Dako, Santa Clara, CA, USA) for total T cells, CD4 (DF16080, Affinity Biosciences, Cincinnati, OH, USA) for T-helper cells, CD8 (IR62361-2, Dako) for cytotoxic T cells, CD14 (E-AB-71017, Elabscience, Houston, TX, USA, diluted 1:200) for monocytes, CD68 (IS61330-2, Dako) for macrophages, CD56 (IR62861-2, Dako) for natural killer cells, and CD79α (IS62130-2, Dako) for B cells. Antibody specificity was verified using positive control tissues (cervical carcinoma for p16; human tonsil and lymph node for immune markers) and negative controls processed without primary antibody.

### 2.4. Imaging and Digital Spatial Analysis

Representative regions were imaged using an Olympus CKX41 inverted microscope equipped with an EP50 digital camera (Olympus Europa SE & Co. KG, Hamburg, Germany) at 200× magnification. Images were analyzed using HALO Image Analysis software (version 3.4, Indica Labs, Corrales, NM, USA). For each sample, senescent cells (p16^+^) and immune cells were segmented and quantified, and senescent cell density (cells/mm^2^) and distribution patterns were evaluated.

Cell quantification was restricted to the stromal compartment; percentages were calculated relative to the total number of stromal nuclei, excluding glandular epithelium and vascular structures. For each marker, the number of positive cells was divided by the total number of stromal nuclei per analyzed field, and the results were expressed as the percentage of stromal cells positive for each marker. This normalization ensured comparability between samples with variable stromal density.

For spatial analysis, adjacent serial sections stained for p16^+^ and for individual immune markers were digitally overlaid and aligned in HALO to ensure precise matching of corresponding stromal regions. Nearest-neighbor (NN) analysis was then applied to calculate the distance of each immune cell to the closest p16^+^ cell, following validated approaches for serial-section registration and spatial mapping [15,16].

### 2.5. Statistical Analysis

Continuous data are presented as mean ± standard deviation (SD) or median (range), depending on distribution. Senescent and immune cell abundances were expressed as percentages of stromal cells. Nearest-neighbor distances were summarized as mean ± SD. Paired comparisons of distances between senescent and different immune subsets were performed using paired *t*-test or the non-parametric Wilcoxon signed-rank test, as appropriate. Correlations between senescent cell density and nearest-neighbor distances to immune subsets were assessed with Spearman’s rank-order correlation coefficient (ρ). A *p*-value < 0.05 was considered statistically significant. Statistical analyses were performed using SPSS software, version 21.0 (SPSS Inc., Chicago, IL, USA).

## 3. Results

### 3.1. Patient Characteristics

A total of 68 women undergoing IVF treatment were included in the study. The mean age was 34.8 ± 6.2 years, and the mean body mass index (BMI) was 23.0 ± 3.4 kg/m^2^. Biopsies were obtained during the mid-luteal implantation window. The main indications for IVF treatment were tubal factor infertility (43%) and unexplained infertility (57%). None of the patients had a history of autoimmune disease, oncological disease, or chronic inflammatory conditions.

### 3.2. Abundance of Senescent and Immune Cells in the Endometrium

**Figure 2:**
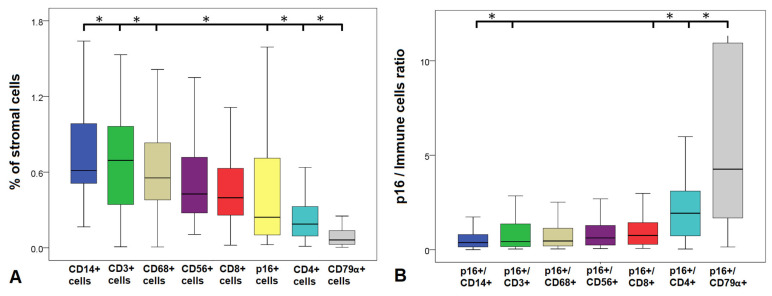
Relative abundance and ratios of senescent and immune cells in the endometrial stroma. Box plots showing: (**A**) the percentage of stromal cells positive for senescent (p16^+^) and immune markers. Horizontal lines with asterisks indicate statistically significant differences between abundances assessed by pairwise comparison (paired non-parametric test, *p* < 0.05). (**B**) Ratios of p16^+^ cells to immune subsets. Asterisks denote statistically significant differences (* *p* < 0.05).

Quantitative analysis revealed differences in the relative abundance of senescent and immune cell populations in the endometrial stroma (Figure 2A). The median percentage of senescent (p16^+^) cells was 0.30% (range 0–12.65). Among immune subsets, monocytes (CD14^+^) represented the largest population, followed by T cells (CD3^+^) and macrophages (CD68^+^). B cells (CD79α^+^) were the least abundant.

Statistical comparison showed that the proportion of senescent cells was significantly lower than that of CD3^+^ (*p* = 0.04), CD14^+^ (*p* = 0.01), and CD68^+^ cells (*p* = 0.01), but significantly higher than the proportion of CD4^+^ T-helper cells (*p* = 0.01) and B cells (*p* = 0.01). No significant differences were observed between p16^+^ cells and CD8^+^ (*p* = 0.76) or CD56^+^ NK cells (*p* = 0.28).

A detailed summary of the quantitative data for all analyzed markers, including median values and observed ranges, is provided in Table 1.

### 3.3. Ratios of Senescent to Immune Cells

To account for inter-individual variability in stromal cellularity, we next calculated the ratios of senescent (p16^+^) cells to specific immune cell subsets (Figure 2B). The ratios were largely similar across most immune populations, with median values close to 1. Lower ratios were observed for comparisons with monocytes (p16^+^/CD14^+^), total T cells (p16^+^/CD3^+^), macrophages (p16^+^/CD68^+^), NK cells (p16^+^/CD56^+^), and cytotoxic T cells (p16^+^/CD8^+^), consistent with the greater abundance of these subsets. In contrast, ratios of p16^+^/CD4^+^ and p16^+^/CD79α^+^ were significantly higher (*p* < 0.05, pairwise test), due to the relatively low frequency of T-helper and B cells in the endometrial stroma. These quantitative and ratio-based analyses prompted us to next investigate how senescent cells are spatially arranged within the endometrial stroma.

### 3.4. Distribution of Senescent (p16^+^) Cells in the Endometrium

Beyond their relative abundance, we next assessed how p16-positive senescent cells were spatially distributed within the functional layer of the endometrium. Stromal p16-positive senescent cells were identified in all studied samples, with a mean density of 291 ± 391 cells/mm^2^ (median 186 cells/mm^2^, range 0–12,698). The high standard deviation and broad range indicate substantial inter-patient variability. Within individual biopsy sections, p16^+^ cell density also varied markedly across different stromal areas, with distinct regions showing either sparse or more densely populated stromal compartments, thus illustrating well-pronounced intra-sample heterogeneity.

**Figure 3:**
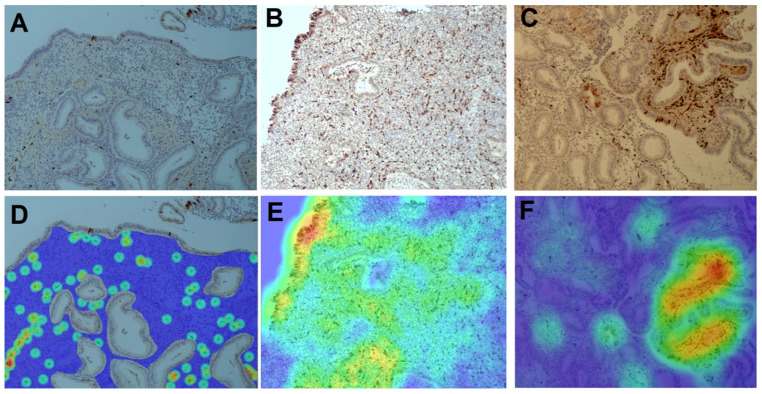
Visualization of spatial distribution patterns of p16-positive senescent cells in the endometrium using conventional and computational analysis. (**A**–**C**) Immunohistochemical staining for p16 shows three representative patterns of stromal distribution: (**A**) low density, (**B**) high density, and (**C**) clustered accumulation. (**D**–**F**) Corresponding heatmap visualizations of the same regions generated with HALO software (version 3.4, Indica Labs, Corrales, NM, USA): (**D**) low-density region, (**E**) high-density region, and (**F**) focal cluster. Warm colors (red/yellow) indicate areas with high numbers of p16^+^ cells, while cooler colors (green/blue) represent regions of lower density.

In most cases, senescent cells appeared as isolated or scattered elements, without forming discernible aggregates (Figure 3A,B). Spatial pattern analysis confirmed that the majority of samples followed a dispersed or near-random distribution model. In a minority of cases (5.2%), localized high-density clusters were detected, with focal areas reaching densities above 10,000 cells/mm^2^ (Figure 3C). Heat map visualization corroborated these findings by highlighting areas of both low and high cell concentration within the same tissue sections (Figure 3D–F). Taken together, these data demonstrate that senescent cells are generally present at low overall density in the endometrium, with occasional focal accumulations but rare clustering events.

### 3.5. Spatial Relationships Between Senescent and Immune Cells

**Figure 4:**
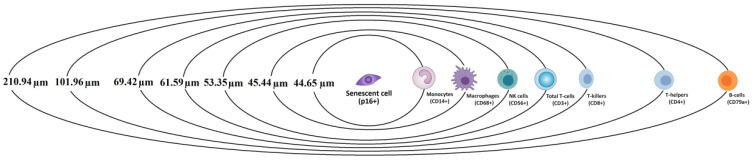
A schematic representation of the results from the performed nearest neighbor analysis. The average distances from the studied immune cells to the nearest senescent cell.

Nearest-neighbor analysis revealed distinct differences in the proximity of immune cell subsets to senescent (p16^+^) cells (Figure 4). Macrophages (CD68^+^) and monocytes (CD14^+^) were positioned closest to senescent cells, with mean distances of 45 ± 20 μm and 45 ± 25 μm, respectively. NK cells (CD56^+^) and total T cells (CD3^+^) were located at intermediate distances (53 ± 23 μm and 62 ± 29 μm). In contrast, T-helper cells (CD4^+^) were positioned significantly farther away (102 ± 42 μm; *p* < 0.05), while B cells (CD79α^+^) displayed the greatest separation from senescent cells (211 ± 66 μm; *p* < 0.01).

A schematic representation of these average distances highlights the selective spatial organization of immune populations around senescent cells, with myeloid subsets positioned in closer proximity and lymphoid subsets—particularly CD4^+^ T cells and CD79α^+^ B cells—showing marked distancing (Figure 4).

**Figure 5:**
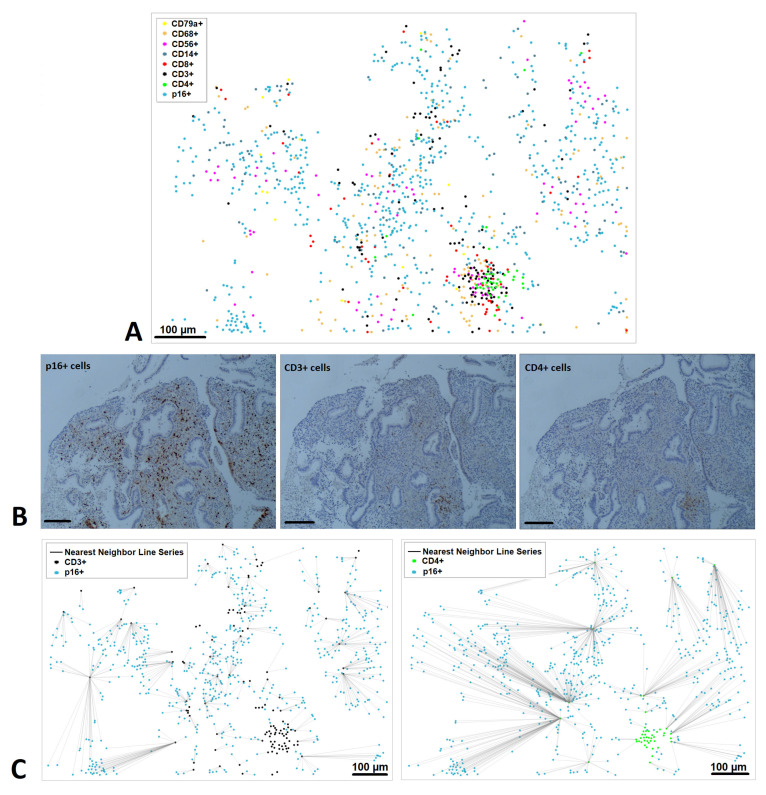
Microphotographs of representative region of the functional layer of immunohistochemically stained human endometrium. (**A**) Distribution map with eight-color scale image. Scale bar, 100 µm. Colored dots representing the individual senescent and immune cells, showing the location of each cell in the image. (**B**) Corresponding IHC images; Expression of p16 (blue), CD3 (black) and CD4 (green) in the stroma. (**C**) Spatial distribution and nearest neighbor line series between p16^+^ cells and CD3^+^ cells and between p16^+^ cells and CD4^+^ cells in the same tissue area.

To further illustrate the analysis, representative images from the functional layer of the endometrium are shown in Figure 5. Distribution maps of multiple immune subsets confirmed the heterogeneous localization of senescent and immune cells (Figure 5A). Immunohistochemical staining demonstrated the presence of p16^+^, CD3^+^, and CD4^+^ cells within adjacent stromal regions (Figure 5B). Nearest-neighbor plots validated the spatial patterns, showing direct positioning of senescent cells relative to CD3^+^ T cells, but increased separation from CD4^+^ T-helper cells (Figure 5C).

### 3.6. Correlation Between Senescent Cell Density and Spatial Distances

**Figure 6:**
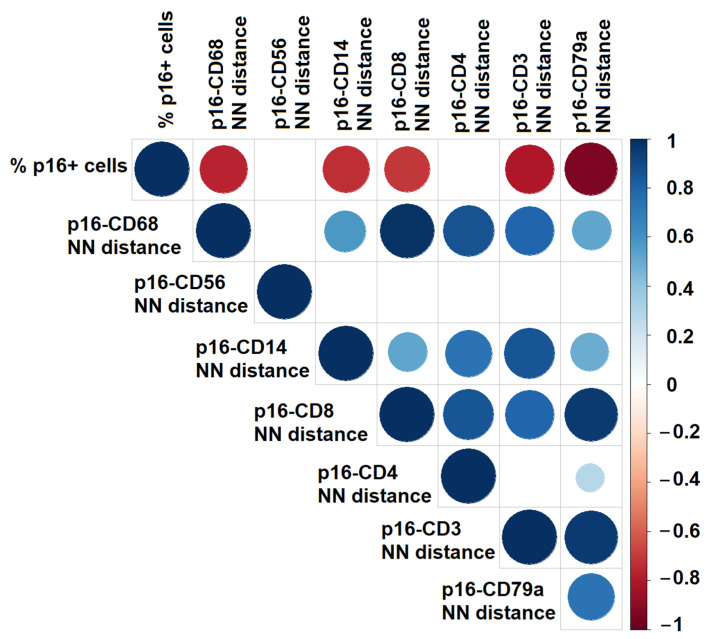
Spearman correlation analysis between the percentage of senescent (p16^+^) stromal cells and nearest-neighbor (NN) distances from p16^+^ cells to individual immune cell subsets. The correlogram illustrates the strength and direction of correlations: blue circles indicate positive associations and red circles negative associations. Circle size and color intensity are proportional to the magnitude of the correlation coefficient (ρ). Empty grid cells represent non-significant correlations.

The spatial distances between p16^+^ senescent cells and immune subsets generally correlated with the relative abundance of immune cells, suggesting that proximity was largely density-driven (ρ > 0.30, *p* < 0.05). This trend was confirmed by Spearman correlation analysis (Figure 6), which showed consistent positive associations among most NN distances. In contrast, two subsets deviated from this pattern. Distances between senescent cells and CD4^+^ T-helper or CD56^+^ NK cells remained relatively large irrespective of immune cell proportion (ρ = 0.26 and ρ = 0.47, ns), indicating an exclusionary rather than density-driven effect. Moreover, in contrast to the strong inter-correlations observed among other NN distances, p16–CD56 and, to a lesser extent, p16–CD4 distances showed weak or absent correlations with the remaining subsets. This unique lack of concordance further supports the notion that NK and T-helper cells are selectively excluded from senescent niches.

## 4. Discussion

In this study, we provide a detailed spatial characterization of senescent (p16-positive) cells in the functional layer of the human endometrium during the implantation window. Our findings demonstrate that senescent cells are heterogeneously distributed throughout the stroma, with occasional high-density clusters. Furthermore, senescent cells displayed selective spatial relationships with immune populations: macrophages and monocytes localized in close proximity, whereas T-helper and NK cells were positioned at significantly greater distances.

The heterogeneous distribution of p16^+^ cells is consistent with previous reports suggesting that endometrial senescence is a physiological process contributing to decidualization and tissue remodeling [4,6]. However, focal clusters of senescent cells may reflect localized dysregulation of senescence clearance. Similar clustering phenomena have been described in pathological contexts such as fibrotic lung disease, where senescent fibroblasts accumulate and disrupt tissue homeostasis [17]. Comparable spatial heterogeneity of senescent niches has also been reported in aging endometrium and other reproductive tissues [18,19,20]. The presence of such clusters in the endometrium may thus indicate imbalance between induction and immune-mediated removal of senescent cells.

A particularly intriguing observation was the non-random spatial distancing of CD4^+^ T-helper and CD56^+^ NK cells from senescent cells. This pattern persisted regardless of immune cell density, as confirmed by correlation analysis, suggesting an exclusionary rather than density-driven effect. NK cells are known to clear senescent decidual cells via granzyme-mediated senolysis [4,21], and their reduced proximity to p16^+^ niches may reflect impaired immune surveillance or altered chemokine guidance. Similarly, CD4^+^ T cells, which are essential for shaping the local cytokine milieu, showed limited spatial association with senescent cells, potentially contributing to an altered immunological environment. Interestingly, this trend was also reflected in the correlation analysis, where p16–CD56 and, to a lesser extent, p16–CD4 distances failed to correlate with other immune subsets, reinforcing their distinct spatial behavior. Analogous immune evasion patterns have been reported in tumor microenvironments, where senescent or tumor-associated cells establish “immune deserts” that limit effector recruitment [22,23]. These findings are consistent with emerging studies in reproductive medicine showing that specific NK and T-cell subsets, and their relative positioning, correlate with implantation outcomes [12,24] and are further supported by recent work showing that endometrial immune profiles (incl. NK subsets) can predict FET outcomes [25].

Beyond absolute percentages, we also evaluated the ratios of senescent to immune cells. These ratios were relatively stable across most subsets, reflecting a balance between senescent and immune populations in the endometrium. The exceptions were p16^+^/CD4^+^ and p16^+^/CD79α^+^ ratios, which were significantly higher due to the low abundance of T-helper and B cells. Such ratio-based assessments may provide a complementary diagnostic dimension, highlighting quantitative imbalances that may escape notice when only absolute percentages are considered. Previous work has similarly shown that altered immune ratios, including NK/T and macrophage subtypes, correlate with implantation success or failure [12,13,26]. Importantly, immune cells not only differ in abundance but also undergo functional shifts during and after implantation, with recent evidence showing that uterine macrophages and NK cells adapt their gene expression while maintaining pro-invasive properties that support trophoblast invasion [27].

Importantly, our methodological approach relied on alignment of adjacent serial sections for spatial analysis. This digital overlay strategy allowed precise matching of stromal regions between senescent and immune markers, enhancing the reliability of nearest-neighbor measurements. While limited to two-dimensional imaging, this approach establishes a reproducible framework for spatial mapping of endometrial cell populations and can be extended to other tissue contexts.

Taken together, our findings reveal that senescent cells might actively contribute to the spatial organization of the endometrial immune landscape. Rather than being randomly distributed, they establish proximity with monocytes and macrophages while excluding NK and T-helper cells. These exclusionary patterns resemble immune-evasion phenomena in other pathological contexts and may represent early signs of impaired receptivity. Importantly, advances in spatial transcriptomics and multiplex imaging now allow the integration of quantitative and spatial data [28,29], offering opportunities to validate and expand our observations. Positioning-based biomarkers, rather than cell counts alone, could thus emerge as clinically relevant tools for assessing endometrial readiness for implantation.

While our study provides new insights into the spatial organization of senescent and immune cells in the endometrium, several methodological considerations should be noted. First, spatial analysis was based on two-dimensional immunohistochemistry, which cannot fully capture the three-dimensional architecture of the tissue. Second, we focused on a limited set of immune markers; additional subsets and functional markers (e.g., markers of activation states, cytokine expression) would add valuable insight. Finally, the cohort consisted of IVF patients, but implantation outcomes were not analyzed because the primary aim was to establish a descriptive spatial framework. Future studies integrating spatial transcriptomics, multiplex proteomics, and clinical outcomes will be critical to validate the diagnostic potential of these observations.

## 5. Conclusions

Senescent p16-positive cells in the human endometrium are heterogeneously distributed, with occasional focal clusters of high density. Spatial analysis demonstrated selective associations with immune subsets: monocytes and macrophages localized in closest proximity, while T-helper and NK cells were positioned at significantly greater distances, independent of their abundance. Ratio-based analyses further highlighted relative enrichment of senescent cells over T-helper and B cells, reflecting quantitative imbalances in specific immune compartments.

These findings indicate that senescent cells actively shape the local immune microenvironment through both quantitative and spatial mechanisms. The non-random exclusion of T-helper and NK cells from senescent niches suggests impaired immune–senescence crosstalk. Spatial profiling of senescent cells may therefore provide novel insights into endometrial biology and holds potential as a diagnostic tool for assessing endometrial receptivity in IVF patients.

## Figures and Tables

**Table 1:** Percentage of senescent and immune cells in the endometrial stroma in the studied women.

Cell Population	Median (Min–Max)	Senescent/Immune Cell Ratios	Median (Min–Max)
p16^+^ cells (Senescent cells), %	0.29 (0–12.65)		
CD3^+^ cells (T-cells), %	0.65 (0.01–20.88)	p16^+^/CD3^+^	0.49 (0–100.00)
CD4^+^ cells (T-helpers), %	0.19 (0.00–4.05)	p16^+^/CD4^+^	1.93 (0.04–25.51)
CD8^+^ cells (T-killers), %	0.38 (0.02–9.85)	p16^+^/CD8^+^	0.81 (0–5.98)
CD56^+^ cells (NK cells), %	0.44 (0.11–13.15)	p16^+^/CD14^+^	0.39 (0–8.56)
CD14^+^ cells (Monocytes), %	0.69 (0.04–17.14)	p16^+^/CD68^+^	0.52 (0–118.43)
CD68^+^ cells (Macrophages), %	0.56 (0.01–14.44)	p16^+^/CD56^+^	0.72 (0–11.00)
CD79α^+^ cells (B-cells), %	0.06 (0.01–5.57)	p16^+^/CD79α^+^	4.74 (0–119.27)

Data as median and range (Min–Max) in parenthesis.

## Data Availability

The data presented in this study are available on request from the corresponding author due to patient privacy and confidentiality reasons.
